# Claudins proteins in brain tumors: expression patterns and therapeutic target

**DOI:** 10.11613/BM.2026.010501

**Published:** 2025-12-15

**Authors:** Adrianna Romanowicz, Marta Łukaszewicz-Zając, Barbara Mroczko

**Affiliations:** 1 Department of Biochemical Diagnostics, Medical University of Bialystok, Bialystok, Poland; 2 Department of Neurodegeneration Diagnostics, Medical University of Bialystok, Bialystok, Poland

**Keywords:** claudins, biomarkers, glioblastoma, central nervous system neoplasms, blood-brain barrier

## Abstract

Tight junctions (TJs) are essential for preserving cell polarity and controlling permeability. It has been disclosed that TJ proteins, especially specific claudins (CLDNs), are linked to inflammation and contribute to the emergence of diverse cancers, including brain malignancies. Aggressive gliomas, including glioblastoma multiforme (GBM), remain among the most common and deadly central nervous system (CNS) tumors worldwide, despite considerable advances in diagnostic and therapeutic approaches. These types of tumors are characterized by high rates of recurrence and metastasis, resulting in poor outcomes and prognosis. The pathophysiology of brain cancer is closely linked to CLDNs, as these specific proteins play critical roles in tumor cell proliferation, invasion, and disruption of the blood-brain barrier (BBB). Some studies reported the potential role of CLDNs in glioma progression and other neurological disorders. The purpose of this review is to highlight the significance of CLDNs in CNS tumors, especially their participation in the formation of malignant gliomas. Additionally, the diagnostic and prognostic importance of selected CLDNs has been assessed. Selected CLDNs, such as CLDN3 and CLDN4 promote GBM growth, proliferation and migration. Moreover, overexpression of CLDN3 support progression and metastasis of these malignancies, while reduced expression of CLDN1 and CLDN5 is observed in advanced gliomas. Presented results suggest that CLDNs may serve as biomarkers for diagnosis and prognosis as well as therapeutic targets in CNS tumors. Further investigation is essential to clarify their clinical relevance and therapeutic potential.

## Graphical abstract

**Figure:**
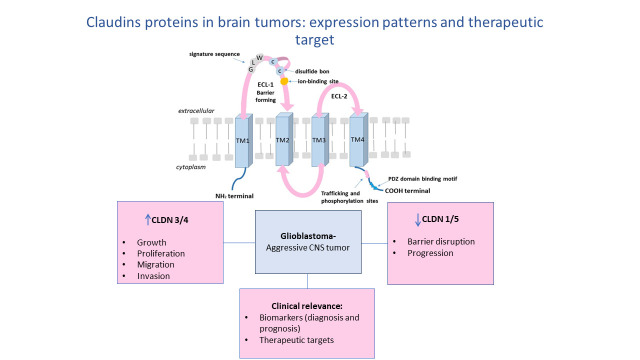

## Highlights

• Claudins contribute to the aggressive growth and metastasis of gliomas

• Claudin-3 and claudin-4 overexpression promotes glioblastoma progression

• Reduced claudin-1, claudin-5, and claudin-6 expressions are observed in advanced gliomas

• Claudin-4 is linked to larger tumor size and may serve as a prognostic marker

• Claudins hold potential as diagnostic biomarkers and therapeutic targets for central nervous system tumors

## Introduction

The significance of selected claudins (CLDNs) that play a crucial role in regulating the paracellular permeability and maintaining the polarity of epithelial cells has been studied extensively in many malignancies, including tumors of the central nervous system (CNS) (*1*-*3*). Certain researchers propose that these proteins could serve as promising biochemical indicators in the development of CNS tumors. Consequently, this review aims to provide an in-depth analysis of the expression patterns, functional significance, and biomarker potential of selected CLDNs in CNS malignancies, with particular emphasis on glioblastoma (GBM), the most prevalent and aggressive form of glioma. The paper highlights the emerging role of claudins in tumor progression, invasion, and therapeutic resistance, emphasizing their biological relevance in CNS tumor development. As a narrative review, it outlines the expression profiles of selected CLDNs in various CNS tumors and explores their potential as biochemical biomarkers for diagnosis and prognosis. A more comprehensive understanding of CLDN activity in brain tumors could thus provide critical insights into the molecular mechanisms driving CNS malignancies and guide the development of more effective therapeutic strategies. Moreover, the therapeutic implications of targeting CLDNs are also discussed. Numerous clinical studies have demonstrated that disruptions in tight junctions (TJs), primarily composed of claudins, are closely associated with malignant transformation. Claudins play a pivotal role in regulating tumor invasiveness, proliferation, and metastasis, positioning them as key elements in brain cancer pathophysiology and as promising therapeutic targets (*2*, *3*).

## Methods of literature search

In preparing this review, a comprehensive literature search was conducted using PubMed, Scopus, and Web of Science databases. Only peer-reviewed articles published in English were considered. The key terms used in the literature search included “Claudins”; “Biomarkers”; “Glioblastoma”; “Central Nervous System Neoplasms”; “Blood-Brain Barrier”; “Tight Junctions”. The primary focus was on literature published between 2010 and 2024. Nevertheless, due to the limited number of recent original studies addressing general characteristics of claudins, older publications were also included where relevant and scientifically valuable. In line with the principles of the San Francisco Declaration on Research Assessment (DORA), an effort was made to cite primarily original research articles to emphasize the value of primary scientific data. However, selected review articles were also included to provide broader context, summarize current understanding, and support the interpretation of original findings.

## Basic molecular features of the claudins protein family

Claudins constitute a family of transmembrane proteins comprising 26 members in humans and 27 in rodents, with molecular weights ranging from approximately 20 to 34 kDa (*4*). These proteins are grouped into two categories based on their sequence similarity: classic claudins (CLDNs1-10, CLDN14, CLDN15, CLDN17, CLDN19) and non-classic claudins (CLDNs11-13, CLDN16, CLDN18, CLDNs20-24) (*1*-*5*). Claudins are part of a multi-gene family, with several closely related pairs of CLDN genes located near each other in the human genome. Functionally, these proteins can be further categorized into barrier-forming claudins (such as CLDN1 and CLDN5) and pore-forming claudins (like CLDN2, CLDN10, and CLDN15) (*1*-*4*). Chromosomal localization and functional roles of classic and non-classic claudins have been presented in Table 1.

**Table 1:** Chromosomal localization and functional roles of classic and non-classic claudins

**Category**	**Claudin**	**Chromosome location of the gene**	**General function**	**References**
	CLDN1	3q22.3		
	CLDN2	Xq22.3		
	CLDN3	7q11.23		
	CLDN4	7q11.23		
Classic claudins	CLDN5	22q11.21	Classical claudins are a family of tight junction proteins primarily involved in maintaining the integrity and selective permeability of epithelial and endothelial barriers	(1-9)
	CLDN6	16p13.3		
	CLDN7	17p13.1		
	CLDN8	21q22.11		
	CLDN9	16p13.3		
	CLDN10	13q32.2		
	CLDN11	3q26.2		
	CLDN12	7q21.11		
	CLDN13	8q22.1		
	CLDN14	21q22.3		
	CLDN15	7q21.11		
	CLDN16	3q28		
	CLDN17	21q22.11		
Non-classic claudins	CLDN18	3q22.3	Non-classical claudins differ from their classical counterparts in that they often exhibit more diverse and specialized functions beyond the formation of tight junctions. Their primary roles include: modulation of tumor microenvironment, tissue-specific expression, regulation of signaling pathways	(1-9,46-49)
	CLDN19	1p34.2		
	CLDN20	6q25.3		
	CLDN21	4q35.1		
	CLDN22	4q35.1		
	CLDN23	8p23.1		
	CLDN24	4q35.1		
	CLDN25	11q23.1		
	CLDN26	8q24.3		
	CLDN27	21q22.11		

**Figure 1:**
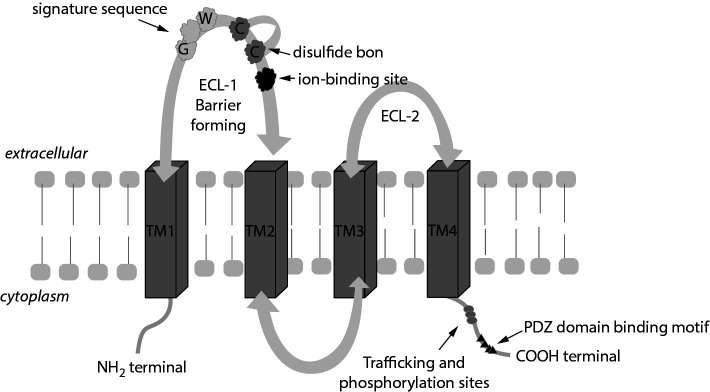
Structure of claudin proteins. Claudins consist of an N-terminal cytoplasmic segment, four transmembrane (TM) regions, two extracellular loops (ECL-1 and ECL-2), and a C-terminal cytoplasmic tail. They form channels that selectively allow ions to pass between cells, with the shorter extracellular loop adopting a helix-turn-helix configuration. Two cysteine residues form internal disulfide bonds, maintaining protein stability. The C-terminal tail contains a PDZ-domain-binding motif, enabling interactions with cytoplasmic tight junction components. Positioning and function of claudins are also regulated by phosphorylation. COOH terminal - carboxy-terminal domain. NH_2_ terminal - amino-terminal domain. ECL-1 - extracellular loop 1. ECL-2 - extracellular loop 2. TM1-4 - transmembrane domains 1 to 4. Figure was modified and adopted from reference (*3*).

Claudins play a crucial role in regulating the paracellular permeability and maintaining the polarity of epithelial cells by forming TJs (*2*-*4*). Tight junctions consists of integral transmembrane (TM) proteins and peripheral membrane proteins that engage in intricate protein-protein interactions. They include endogenous transmembrane proteins such as CLDNs and occludin, as well as cytoplasmic proteins like *zonula occludens*, which connect the actin cytoskeleton and signaling proteins (*3*). Claudins are made up of an N-terminal segment in the cytoplasm, four transmembrane regions, two extracellular loops, and a C-terminal cytoplasmic tail (*1*-*4*, *6*). These proteins exhibit a structural pattern that includes four predicted TM regions, with one larger extracellular loop containing a conserved sequence motif, and a second, shorter extracellular loop (*1*-*4*, *6*). Claudins also create channels that selectively allow certain ions to pass between cells, while the shorter extracellular loop displays a helix-turn-helix configuration (*7*). Figure 1 illustrates the structure of claudin. Additionally, two cysteine residues form internal disulfide bonds, playing a key role in maintaining the protein’s stability (*6*). The C-terminal tail of claudins shows variability in both sequence and length, and it includes a PDZ-domain-binding motif (PSD95 - postsynaptic density protein; Dlg1 - Drosophila disc large tumor suppressor; ZO-1 - *zonula occludens*-1 protein), which enables direct interactions with cytoplasmic components of TJs. The positioning and role of CLDNs are also controlled by the phosphorylation of the C-terminal, which is a target of serine, threonine, and tyrosine kinases (*6*).

Claudins modulate the functional alterations of TJ protein through post-translational changes. Processes like phosphorylation, ubiquitination, palmitoylation, and glycosylation as post-translational modifications impact CLDNs structure, stability, transport, and function (*8*). Research by Suzuki *et al*. has demonstrated that the transmembrane segments of mammalian claudin 15 (mCLDN15) create a characteristic left-handed four-helix bundle (*9*). Moreover, significant portions of the two extracellular segments form a distinct β-sheet fold, encompassing two extracellular segments. These findings suggest that the linear arrangement of mCLDN15 protomers observed in the lipidic cubic phase (LCP) crystals might be indicative of linear claudin polymers that are formed in TJs. The authors conclude that understanding the crystal structure of CLDNs, which are the building blocks of TJ strands, will enhance our molecular understanding of the paracellular barriers between epithelial cells in multicellular organisms (*9*). The primary role of most members of the CLDN is to establish paracellular barriers, regulate intramembranous diffusion, and maintain cellular polarity (*10*, *11*). These proteins are predominantly localized in the apical membrane region, where they contribute to the formation of TJ complexes, essential for preserving both cell polarity and intercellular adhesion. Consequently, CLDNs are found to be critical for the proper functioning of the intercellular barriers formed by TJs, underscoring their significance in cell–cell junction integrity and tissue homeostasis (*11*).

## Claudins and cancer pathogenesis

In addition to forming the structural backbone of tight junctions, claudins play pivotal physiological roles in regulating paracellular permeability and maintaining epithelial cell polarity. Their characteristic structure enables them to function both as barriers and selective pores. Moreover, claudins can directly modulate signaling pathways or indirectly influence cell growth, survival, proliferation, and differentiation through interactions with other proteins. Their expression is tightly regulated and varies across tissues, with additional diversity introduced by alternative splicing. In cancer, the disruption of normal epithelial architecture compromises tight junction integrity, affecting the function and localization of claudins (*12*). While these proteins are vital for maintaining the epithelial barrier, their significance in neoplasia is not fully understood (*12*). The loss of claudins disrupts the integrity of cell connections, one of the characteristics of epithelial-mesenchymal transition (EMT). In cancer, the loss of cell polarity and differentiation is closely tied to metastasis, with EMT serving as a crucial mechanism in this progression (*12*). The process of EMT starts with the disintegration of the connection among epithelial cells, including adherens junctions, TJs, desmosomes, and gap junctions, which can lead to a disruption of cell polarity and reorganization of the cytoskeleton. Claudins, as the proteins associated with TJs, have been found to contribute to various stages of cancer progression (*12*). An increasing amount of research indicates that tumor growth is influenced not only by intrinsic mechanisms but also by paracrine signals, including specific growth factors secreted by the TM. Such signaling molecules are typically produced as transmembrane proteins and get released through a process of limited proteolysis known as ectodomain shedding (*13*). Claudins expression and localization are frequently altered in a variety of tumors, which can facilitate cancer progression by impairing cell polarity and increasing cellular migration. Claudin-1 has been shown to be highly expressed in gastric cancer tissues, particularly in intestinal-type tumors and CLDN1 overexpression was positively associated with more advanced TNM stages, the presence of lymph node metastasis and well-differentiated tumors. Its elevated expression also appears to be negative prognostic factor, especially in the intestinal-type of gastric cancer (*14*). Claudin-1 primarily acts as a tumor promoter by enhancing the invasive and motility capabilities of cancer cells. This effect is mediated through its role in altering cell adhesion, disrupting epithelial barrier integrity EMT, a key process in cancer metastasis. Additionally, CLDN1 has been implicated in modulating signaling pathways, such as Wnt/β-catenin, further contributing to intestinal-type gastric cancer progression and the spread of malignant cells (*14*). Quantitative real-time polymerase chain reaction (qRT-PCR) analysis revealed that CLDN1 mRNA expression was significantly elevated in non-small cell lung cancer (NSCLC) tissues compared to adjacent normal tissues. In addition, CLDN1 mRNA expression was significantly higher in the tissue of NSCLC patients in advanced TNM stages (III–IV) and with the presence of lymph node metastases in comparison to subjects without lymph node metastasis and at stages I and II, what indicate the role for CLDN1 in the progression and metastasis of NSCL (*15*). Additionally, CLDN2 displays elevated expression in human lung adenocarcinoma cells and contributes to enhanced cell proliferation, while its knockdown has been shown to increase paracellular permeability and intracellular accumulation of doxorubicin in tumor spheroids. These findings suggest that loss of CLDN2 expression may enhance the sensitivity of lung adenocarcinoma cells to anticancer agents (*16*). In the study of Okui *et al.* several clonal populations representing aggressive and non-aggressive phenotypes from a human pancreatic cancer cell line were isolated and subsequently authors identified CLDN7 as a gene specifically expressed in the clonal population with aggressive phenotypes. DNA microarray analysis revealed a high expression of CLDN7 in the human pancreatic cell lines MIA PaCa-2-A (cells with an epithelial morphology) compared to low expression of CLDN7 in the MIA PaCa-2-R cells (with a non-epithelial morphology), while the knockdown of CLDN7 in the MIA PaCa-2-A cells induced a marked inhibition of proliferation (*17*). Authors conclude that CLDN7 is expressed in the rapidly proliferating and dominant cell population of pancreatic cancer tissues and represents a potential molecular target for pancreatic cancer treatment (*17*).

## Classification of central nervous system tumors

Central nervous system tumors account for roughly 2% of all cancer cases globally (*18*). Such tumors belong to a highly diverse category of growth, characterized by distinct disease progressions and varying patient outlooks. According to the 2021 World Health Organization (WHO) classification, tumors of the CNS are now categorized based on a combination of histological grading (grades 1 to 4) and molecular characteristics, such as isocitrate dehydrogenase (IDH) mutation status, 1p/19q co-deletion, and telomerase reverse transcriptase (TERT) promoter mutations (*18*). Gliomas, tumors of glial origin, are the most common primary malignant CNS tumors, accounting for over 40% of cases. Glioblastoma multiforme, which accounts for 55% of all neuroepithelial tumors in adults, remains the most lethal CNS malignancy. The pathogenesis of CNS tumors, including gliomas, involves a combination of genetic and epigenetic alterations. Advances in understanding these processes have paved the way for novel therapies targeting cancer stem cells and epigenetic regulators. Several environmental factors, such as alcohol and tobacco use, mobile phone usage, contact with chemical substances, head trauma, infections, injuries, N-nitroso compounds, and low-frequency electromagnetic fields, have been reported to be potentially associated with the development of brain tumors (*19*-*21*). However, the only well-established risk factor for these neoplasms is prior exposure to high-dose ionizing radiation (*19*-*21*).

## Diagnosis of central nervous system tumors

Imaging modalities play a crucial role in the diagnostic evaluation of CNS tumors (*20*, *21*). Despite significant advancements in both structural and functional neuroimaging techniques, the survival rates for patients remain unsatisfactory. Unfortunately, current neuroimaging methods lack the specificity required for effective differentiation between various types of brain tumors. Extensive research efforts have yielded minimal progress, necessitating that tumor grading and classification should be performed through histopathological analysis of tissue specimens, which inherently involve surgical procedures (*20*, *21*). Future diagnostic strategies should focus on integrating molecular biomarkers with histopathological analysis to improve the accuracy and personalization of tumor classification. The continued advancement of molecular diagnostic tools is expected to significantly enhance the early detection, and prognosis of malignant CNS neoplasms, potentially reducing the need for invasive surgical procedures in the future. The introduction of readily available biochemical markers could greatly enhance the diagnostic process for these tumors, facilitating disease monitoring, differential diagnosis, and surgical planning, especially in the early stages of the disease (*22*). In standard clinical practice, the determination of the concentrations of the alpha-fetoprotein (AFP) and beta subunit of human chorionic gonadotropin (β-HCG) are the primary biochemical tumor markers utilized for diagnosing rare pediatric germ cell tumors. Moreover, numerous researchers have investigated the plasma concentrations of various biomarkers in plasma, *e.g.* glial cell line-derived neurotrophic factor (GDNF), brain-derived neurotrophic factor (BDNF), glial fibrillary acidic protein (GFAP), neuropeptide Y (NPY, placental growth factor (PlGF), S100B, and interleukin 8 (IL-8) in patients diagnosed with diverse forms of CNS tumors (*22*). The investigators determined that the plasma concentrations of these biomarkers showed significant correlation with neuropathological classifications and characteristics. Central nervous system tumor markers are valuable, however have several limitations, such as lack of specificity, *e.g.* these substances are not unique to cancer and can be elevated in non-cancerous conditions, leading to false-positive results and unnecessary diagnostic procedures or treatment. Another limitation is lack of sensitivity, *e.g.* might not be elevated in all individuals with cancer or not for all types of cancer, causing false negatives in early stages and missed diagnosis. For example, GFAP which is considered as a prototype glial-specific biomarker, lacks tumor specificity. Elevated GFAP concentration in serum is also observed following stroke or traumatic brain injury, reflecting general brain damage rather than neoplastic transformation (*23*, *24*). Notably, increased serum GFAP concentration after surgical resection occur regardless of glioma grade, confirming its low specificity for tumor burden. Thus, while GFAP may support differential diagnosis or complement imaging findings, its standalone diagnostic value remains limited (*23*, *24*). Similarly, angiogenesis-related proteins have garnered interest due to the role of vascular proliferation in glioblastoma pathogenesis. Vascular endothelial growth factor (VEGF), often elevated in the serum of glioblastoma patients and targeted by therapeutic agents like bevacizumab, is not tumor-specific. Clinical trials evaluating antiangiogenic therapies, such as thalidomide and cediranib, revealed that circulating VEGF concentration neither correlated with overall survival nor reliably reflected disease recurrence (*25*, *26*). This limits its utility as a prognostic or monitoring biomarker in GBM. Epidermal growth factor receptor (EGFR), often amplified or mutated in GBM, showed elevated serum concentration in patients compared to healthy individuals (*26*). However, postoperative concentration did not significantly decline, indicating poor sensitivity to tumor burden changes. Hence, while EGFR alterations are valuable for molecular classification and targeted therapy stratification, serum EGFR concentration alone is not reliable indicators of tumor dynamics (*27*). In summary, while several proteins demonstrate potential as glioma biomarkers, most suffer from limited specificity, inconsistent correlation with tumor progression, and variability due to non-neoplastic brain conditions. Furthermore, comparative evaluation with claudins and other methods like imaging or biopsies, may help to establish more precise diagnostic and prognostic tools in CNS neoplasms. As a result, there is an urgent need for the development of novel biochemical tumor markers. In this context, claudins, owing to their distinct expression patterns and biological functions, have gained attention as potential diagnostic and prognostic biomarkers in CNS malignancies. To provide a concise overview, Table 2 summarizes their expression patterns, functional roles, and clinical significance. The significance of selected CLDNs has also been presented in Table 2.

**Table 2:** The importance of some claudins as potential biomarkers for CNS tumors

**Claudins** **(references)**	**Expression**	**Association with cancer**	**Possible diagnostic/prognostic marker**	**Possible therapeutic target**
CLDN1 (30-33)	Reduced in high-grade gliomas (3 and 4); higher in low-grade gliomas (1 and 2)	Linked to cancer progression and invasiveness	Possible prognostic marker for cancer grading	-
CLDN3 (36,37)	Significantly upregulated in GBM	Supports tumor cell growth and epithelial-mesenchymal transition	-	Potential target *via* TGF-β inhibitors (*e.g*., ITD-1)
CLDN4 (38-41)	Increased with glioma progression	Enhances glioma cell proliferation and migration; correlates with tumor size	Potential biomarker for gliomas	Monoclonal antibody (*e.g.* KM3900) targeting CLDN4 induces cytotoxicity and disrupts tumor tight junctions, enhancing therapy efficacy.
CLDN - claudin. CNS - central nervous system. GBM - glioblastoma multiforme. TGF-β - transforming growth factor-β.				

## Classic claudins in central nervous system malignancies

Claudin-1, the first identified member of the CLDNs, weighing 22 kDa, is highly expressed in organs such as the brain, kidney, liver and spleen (*28*, *29*). In various cancers, CLDN1 shows an altered role; its reduced expression has been linked to cancer progression, increased invasiveness, and the development of metastatic characteristics (*30*, *31*). Investigation highlights CLDN1 as one of the most deregulated claudins in human cancers, acting as either a tumor promoter or suppressor depending on the specific cancer type (*32*). Karnati *et al*. observed a significant reduction in CLDN1 expression in glioblastoma (grade 4) and anaplastic astrocytoma (grade 3) tumor samples, compared to pilocytic astrocytoma (grade 1) and diffuse astrocytoma (grade 2) samples, in which CLDN1 expression was elevated (*33*).

Claudin-3, a member of the transmembrane TJ protein family, serves as a key structural component within TJs (*34*, *35*). Sun *et al.* have reported that CLDN3 is upregulated in GBM and contributes to tumor cell growth and epithelial-mesenchymal transition in both *in vitro* and *in vivo* models (*36*). Their findings suggest that CLDN3 expression can be activated by transforming growth factor-β (TGF-β), while specific inhibitors of the TGF-β signaling pathway, such as inhibitor of TGF-β receptor type I (ALK5) degradation 1 (ITD-1), reduce CLDN3 levels. Further studies showed that elevated CLDN3 levels amplify TGF-β-induced growth and EMT in GBM cells, whereas decreasing CLDN3 diminishes these effects. This research highlights CLDN3 role in supporting GBM growth and metastasis, linking it to the tumor-promoting functions of TGF-β. As a result, targeting CLDN3 with specific inhibitors could offer a promising strategy for treating this aggressive cancer (*36*, *37*).

Claudin-4, a transmembrane protein, is found within the TJs of intestinal epithelial cells and on the surface of M cells in both humans and mice. In microfold cells, CLDN4 functions as a TNF receptor for *Clostridium perfringens* enterotoxin (CPE), interacting specifically with the toxin’s C-terminal 30 amino acids through direct binding (*38*). Elevated CLDN4 expression in glioma tissues are associated with poor clinical outcomes, and Yang *et al.* further demonstrated a positive correlation between CLDN4 expression and the more advanced clinical stages of glioma patients, what was assessed using immunohistochemical staining (IHC) (*38*). *In vitro* investigations demonstrate that elevated CLDN4 expression significantly enhances glioma cell proliferation and migration. Moreover, overexpression of CLDN4 is associated with increased tumor sizes in glioma xenograft models. Additionally, CLDN4 influences glioma progression by modulating the NNAT/Wnt signaling pathway. The tumor-promoting role of the CLDN4/NNAT axis has been further confirmed in glioma organoids. Collectively, these findings suggest that CLDN4 holds promise as a valuable biomarker and a potential therapeutic target for glioma treatment (*38*). Yan *et al*. have found that CLDN4 expression was upregulated in GBM tissues and cell lines compared to paired adjacent normal tissues and normal human astrocytes (NHAs) (*39*). Furthermore, elevated CLDN4 expression were correlated with shorter overall survival in GBM patients. Suppression of CLDN4 expression was shown to reduce mesenchymal transformation, as well as inhibit GBM cell invasion, migration, and tumor growth both *in vitro* and *in vivo.* Functional analyses revealed that CLDN4 is involved in modulating the tumor necrosis factor alpha (TNF-α) signaling pathway. Additionally, the TGF-β pathway was found to upregulate CLDN4 expression and enhance GBM cell invasiveness, while its inhibition by ITD-1 reduced CLDN4 levels and suppressed invasion. TGF-β also promoted the nuclear translocation of CLDN4. Collectively, these results suggest that the TGF-β/CLDN4/TNF-α/NF-κB signaling axis plays a critical role in glioma progression, and its disruption may offer a promising therapeutic strategy for GBM (*39*). Targeting of CLDN4 is expected to provide multi-layered effects by enabling direct attacks on CLDN4-overexpressing cancer cells, disrupting the intratumoral microenvironment, and facilitating drug delivery by impairing TJs. It is also expected to inhibit tumor-promoting signals generated by non-TJ CLDN4. Some clinical investigations generated a monoclonal antibody (KM3900) that targets CLDN4 extracellular loop 2 and induces antibody-dependent cytotoxicity (ADCC) and complement-dependent cytotoxicity (CDC) *in vitro* (*40*). Claudin-4 is expressed in a several normal tissues, and off-target effects of anti-CLDN4 drugs other than antibodies should also be determined (*41*).

Claudin-5 plays a crucial role in regulating the permeability of the blood-brain barrier (BBB) during lung cancer brain metastasis by modulating the proliferation, migration, and permeability of human brain vascular endothelial (hCMEC/D3) cells. This regulation, primarily mediated through the cell adhesion molecule signaling pathway, enhances tight junction integrity and contributes to the suppression of lung cancer brain metastasis formation (*42*). The expression profile of CLDN5 closely corresponded to that of CLDN1, exhibiting similar patterns in both high-grade (*3*, *4*) and low-grade (*1*, *2*) samples (*33*). The main conclusion of the present study is that reduced expression CLDN1 and CLDN5 are linked to the advancement of malignant gliomas, with this effect becoming more evident as the grade of glioma increases. Liebner and colleagues found a frequent reduction in CLDN1 expression in GBM tissue compared to normal brain tissue, without any significant downregulation of CLDN5 (*43*). Likewise, Ishihara and colleagues analyzed 24 cases of both low- and high-grade gliomas and reported a loss of CLDN1 expression in high-grade tumors (*44*). Reduced CLDN1 expression was observed in glioma cell lines derived from high-grade (grade 3 and 4) tumors, compared to low-grade pilocytic astrocytomas (grade 1), diffuse astrocytomas (grade 2), and non-neoplastic control tissues (*43*, *44*).

Claudin-6 shares homology with classical claudins, specifically CLDNs 1–5, 7–10, 14, 15, 17, and 19, distinguishing it from the less homologous non-classical CLDN (*45*). This protein was first identified in 2001 through differential display analysis during the differentiation process of embryoid bodies (EBs) (*46*). Claudin-6 expression is subject to dynamic regulation by multiple factors. It is present in fetal tissues such as the kidneys, pancreas, stomach and, lungs, but notably absent in these tissues in adults (*47*). Claudin-6 also emerges as one of the first proteins expressed during embryonic development in cells as they commit to an epithelial lineage and serves as a surface-specific marker for human pluripotent stem cells (hPSCs) (*48*). The expression profiles of claudins differ across various cancer types, with CLDN6 undergoing extensive investigation through pan-cancer analyses. Claudin-6 was notably upregulated in 20 different cancers; however, its expression was significantly reduced in GBM tissues, chromophobe renal cell carcinoma (KICH), acute myeloid leukemia (LAML), clear cell renal cell carcinoma (KIRC and LGG) (*49*). Moreover, based on research conducted by Zhuang *et al.,* it can be concluded that CLDN10 expression levels vary significantly across cell types (*50*). Claudin-10 is highly expressed in brain endothelial cells, where it contributes to the formation of TJ strands and maintains the integrity and selective permeability of the BBB. Knockdown of CLDN10 in brain endothelial cells significantly reduces transendothelial resistance and increases barrier permeability, facilitating both drug delivery and the transendothelial invasion of breast cancer cells. Importantly, breast cancer cells with low CLDN10 expression showed enhanced ability to cross the brain endothelial barrier, suggesting that CLDN10 downregulation may promote brain metastasis. These findings provide a foundation for further research into potential biomarkers for brain-related diseases (*50*).

## Methodology and technologies applicable to clinical chemistry and laboratory medicine for the evaluation of the claudins

New omics technologies, including proteomics, metabolomics, and lipidomics, enable the identification of biomarkers with high sensitivity, even at the single-cell level (*51*-*53*). In addition, recent advances such as artificial intelligence support the analysis of large heterogeneous datasets, improving diagnostic accuracy by recognizing subtle molecular patterns (*54*). A growing body of evidence indicates that several claudins may serve as potential biomarkers in cancer, although contradictory results have been reported. This suggests that CLDNs can act both as promoters and suppressors of tumor growth depending on context (*55*). The variability in results may arise from differences in sampling strategies and from the lack of standardized approaches for assessing CLDN immunoreactivity and subcellular localization (*55*). Since both the expression level and localization of claudins are critical for maintaining epithelial function, their altered synthesis or mislocalization is characteristic of malignant transformation (*55*). In cancer research, immunochemistry-based methods have been most widely applied to evaluate CLDN expression. Immunohistochemistry (IHC) has been extensively used to assess tissue expression and localization patterns (*14*, *30*). Western blotting has been performed to validate protein concentrations in both tissues and glioma cell lines (*33*). More recently, enzyme-linked immunoassays (ELISA) have been employed to measure circulating CLDN concentrations in patient sera, offering a minimally invasive and cost-effective approach for biomarker evaluation (*56*). For the analysis of mRNA expression, several methods have been used, including real-time RT-PCR to quantify CLDN transcripts in normal and malignant tissues, Northern blotting to assess gene expression profiles, and data mining of the public SAGE database to visualize differential expression (*28*, *29*). Quantitative PCR studies further demonstrated downregulation of CLDN1 and CLDN5 in gliomas of all four grades and in glioma model cell lines (*33*). Finally, DNA-based methods, such as cDNA cloning and sequencing, have been applied to characterize CLDN gene sequences and variants in normal and cancerous tissues (*29*). These approaches provide complementary insights into the genomic basis of altered CLDN expression observed in cancer.

The new omics technologies, including proteomics, metabolomics, and lipidomics are able to identify biomarkers with high sensitivity, even up to individual cell (*51*, *52*). Innovative technologies have been discovered to improve overcome problems, reliability, and enhance the user experience (*53*). Furthermore, artificial intelligence, recently the most investigating issue, is able to evaluate large amounts of heterogeneous data and recognize subtle patterns to answer diagnostic questions (*54*).

Growing body of evidence indicate the role of several claudins as potential biomarkers of cancer disease and reported contradictory results, which demonstrate that these proteins play dual role as promoters as well as tumor-growth suppressors in carcinogenesis (*55*). Some authors indicated that main possible cause of discrepancies in CLDNs levels during neoplastic process is a considerable variability of sampling and the absence of a consistent approach to the evaluation of the immune reactivity of CLDNs and to the differential analysis of subcellular localization of these proteins (*55*). Based on literature data, investigators conclude that only an optimal expression and localization of claudins is important for maintaining the normal physiological function of the epithelium, and any imbalance may have pathological consequences. The synthesis of CLDN in malignant cells as well as their aberrant localization are characteristic for formation of a malignant phenotype (*55*). Therefore, in our present review we presented the investigations, where the levels of CLDNs were assessed mainly by immunohistochemical methods (*14*, *30*). Moreover, we demonstrated the original findings, where authors identified all the human *CLDN* genes and corresponding proteins sequences from GenBank, while to visualize the results the public SAGE database was used to ascertain the gene expression of all *CLDNs* in normal and malignant tissues. In addition, real-time RT-PCR method was employed to explore a subset of CLDN genes in normal and cancerous tissues (*28*). Other methods reported in the study concerning the measurement of CLDNs levels were: Northern Blotting, cDNA Cloning and Sequencing (*29*). Using quantitative PCR and Western blot methods the mRNA and protein status of selected CLDNs genes were studied in human gliomas. Quantitative analysis of the transcript and protein expression data showed that CLDN1, and CLDN5 were significantly down regulated glioma of all four grades, what was also reported in the malignant glioma model cell lines (*33*). Recently, many authors use high sensitivity and specificity, ease of use, and relatively cost-effective enzyme-linked immunoassay (ELISA) method to assess serum concentrations of selected CLDNs in the patients with malignancies as promising biomarkers for predicting the development, proliferative ability, and prognosis of cancer, because blood is easy to obtain and can be obtained repeatedly (*56*).

## Claudins as therapeutic targets in cancer

Two criteria are characteristic for an ideal molecule for targeted therapy in malignancy. Its expression should be restricted in certain tissues, with no expression or inaccessibility to targeting strategies in other normal tissues to avoid adverse effects; as well as the expression of this ideal molecule should be positive, and its epitopes should be exposed in corresponding malignant tissues, rendering them targeted (*57*). Claudins have been discovered to meet the two criteria and as promising targets for the treatment of cancer (*56*). Some clinical investigations proved that normal epithelial cells also express these proteins, but claudin expression has tissue specificity, and some claudins are only expressed in very few tissue types (*56*). Based on this specific expression profile and difference between normal and tumor cells, CLDNs are attractive targets that can theoretically enable selective drug delivery with minimal adverse events. Claudins have been a focus of the biotechnology and pharmaceutical industries as potential therapeutic targets. Moreover, a number of investigations concerning pharmacogenomics allows for assessment of genetic polymorphisms that predict a response to chemotherapeutic agents and reported an idea of personalized or precision medicine by determining susceptibility to certain drugs through identification of genetic variants (*58*). The localization of CLDN1 as a transmembrane protein cause that this chemokine is perfect target for the enhanced drug absorption for preventing infection and treating cancer (*31*). Recently, CLDN1 was successfully targeted with anti-CLDN1 near-infrared fluorophore to track the CRC cells, and it may provide a novel way for fluorescence-guided surgery of tumor. In the future, a similar approach can be applied to CLDN1 by preparation of CLDN1 targeting molecule and can be tested for cytotoxicity to normal cells (*57*). In addition, a chimeric anti-CLDN18.2 monoclonal antibody (mAb) – Zolbetuximab is currently under FDA review that has undergone phase 1, 2, and 3 explorations in advanced CLDN18.2 positive gastric adenocarcinoma tumors (*59*-*61*). Investigators suggest that it may emerge as the first CLDN targeted therapy approved. Other CLDN agents targeting CLDN6 and CLDN4 are ongoing in solid tumors, including mAbs, C-CPE or mAb-drug/material conjugates, bispecific T cell engagers (BiTEs) and chimeric antigen receptor (CAR) T cells are under intensive investigation.

## Conclusions

Numerous clinical studies suggest that selected CLDNs, such as CLDN3 and CLDN4 promote GBM growth, proliferation and migration. Moreover, overexpression of CLDN3 support progression and metastasis of these malignancies, while reduced expression of CLDN1 and CLDN5 is observed in advanced gliomas. The heightened expression of CLDN4 is linked to larger glioma sizes and this claudin might be a promising biomarker and potential therapeutic target of patients with glioma. Moreover, expression of CLDN6 was significantly decreased among others in GBM and LGG. In conclusion, several claudins have the potential to act as prognostic and diagnostic biomarkers, as well as therapeutic targets for CNS tumors. Moreover, further study is necessary to explore innovative approaches that assess the usefulness of these proteins in diagnosing CNS tumors and predicting patient outcomes. Such studies should focus on the varying expression profiles of CLDNs across different tumor histology and disease stages.

## Data Availability

No data was generated during this study, so data sharing statement is not applicable to this article.
